# Exploiting the Potential in Water Cleanup from Metals and Nutrients of *Desmodesmus* sp. and *Ampelodesmos mauritanicus*

**DOI:** 10.3390/plants10071461

**Published:** 2021-07-16

**Authors:** Roberto Braglia, Lorenza Rugnini, Sara Malizia, Francesco Scuderi, Enrico Luigi Redi, Antonella Canini, Laura Bruno

**Affiliations:** 1 Botanic Gardens, Department Biology, University of Rome Tor Vergata, 00133 Rome, Italy; roberto.braglia@uniroma2.it (R.B.); francescoscuderiobtv@gmail.com (F.S.); enrico.luigi.redi@uniroma2.it (E.L.R.); canini@uniroma2.it (A.C.); 2 Laboratory of Biology of Algae, Department Biology, University of Rome Tor Vergata, 00133 Rome, Italy; rgnlnz01@uniroma2.it (L.R.); sara.malizia23@gmail.com (S.M.)

**Keywords:** green microalgae, *Desmodesmus* sp., *A. mauritanicus*, metals, nutrients, bioremediation

## Abstract

Increasing levels of freshwater contaminants, mainly due to anthropogenic activities, have resulted in a great deal of interest in finding new eco-friendly, cost-effective and efficient methods for remediating polluted waters. The aim of this work was to assess the feasibility of using a green microalga *Desmodesmus* sp., a cyanobacterium *Nostoc* sp. and a hemicryptophyte *Ampelodesmos mauritanicus* to bioremediate a water polluted with an excess of nutrients (nitrogen and phosphorus) and heavy metals (copper and nickel). We immediately determined that *Nostoc* sp. was sensitive to metal toxicity, and thus *Desmodesmus* sp. was chosen for sequential tests with *A. mauritanicus*. First, *A. mauritanicus* plants were grown in the ‘polluted’ culture medium for seven days and were, then, substituted by *Desmodesmus* sp. for a further seven days (14 days in total). Heavy metals were shown to negatively affect both the growth rates and nutrient removal capacity. The sequential approach resulted in high metal removal rates in the single metal solutions up to 74% for Cu and 85% for Ni, while, in the bi-metal solutions, the removal rates were lower and showed a bias for Cu uptake. Single species controls showed better outcomes; however, further studies are necessary to investigate the behavior of new species.

## 1. Introduction

The rate of human environmental damage is continuously increasing and represents one of the most urgent challenges we face today. Conventional clean-up techniques, i.e., chemical and engineering-based methods, even if effective, are, in general, very expensive and consist of in-situ and ex-situ interventions, like soil excavation, soil washing or burning, pump and treat systems, solidification with stabilizing agents, vitrification at high temperatures and electrochemical separation. These techniques have some disadvantages considering that they can destroy the soil biotic components and are technically difficult, energy intensive and expensive [1]. 

An efficient alternative to current environmental clean-up methods is phytoremediation, which can be defined as “the use of green plants and their associated microorganisms, soil amendments and agronomic techniques to remove, contain, or render harmless environmental contaminants” [2]. Among the other remediation methodologies, phytoremediation is one of the most cost-effective, environmentally friendly and low energy consumptions and can be accomplished in situ. Plants have many endogenous genetic, biochemical and physiological properties that make them ideal agents for soil and water remediation [3]. 

Various hyper accumulative plant species have been extensively investigated that led to substantial progress in this field [4]. Among the many hyperaccumulator species known today (about 450 angiosperm species) [5], the Brassicaceae family is the most represented one with the genera *Thlaspi*, *Brassica* and *Alyssum*. There are members of the genus *Thlapsi* that are known to accumulate Zn, Cd and Pb. Another important hyperaccumulator species is *Brassica juncea* (L.) Czern., which has a great capacity to absorb heavy metals (HM) and to transport them in the aerial organs, although it does not grow spontaneously on metalliferous soils [4,5,6]. 

Nevertheless, it is important to mention that most hyperaccumulators are relatively small and have slow growth rates [7]. *Ampelodesmos mauritanicus* (Poiret) Dur. Et Sch. is an hemicryptophyte caespitosus belonging to the Poaceae family, which can be 1–2 m high. *A. mauritanicus* form very dense bushes with a 1-m diameter, has erect culms and an angled apex. The flowering period is from April to July. The species has a diffusion through Steno-Mediterranean South-Western Europe and, in Italy, is reported as a spontaneous species in all regions except for Piedmont, Valle d’Aosta, Emilia Romagna, Lombardia, Trentino Alto Adige, Veneto and Friuli Venezia Giulia. It grows along the clay slopes that are generally lapped by humid currents from sea level up to 1200 m [8]. 

Consequently, it is a plant highly resistant to insolation, drought, temperature variability [9]. Algae-based remediation (phycoremediation) techniques exploit the natural ability of cyanobacteria, macro- and microalgae to remove organic/inorganic pollutants (such as nutrients and metals) from wastewater [10,11] when present at concentrations between 1 and 100 mg L^−1^, where conventional methods are insufficient [12,13]. The idea of using microalgae to treat wastewater was introduced in 1957 by Oswald and Gotaas [14], and since then numerous studies have underlined how efficient microalgae remediate highly polluted waters and how they can be incorporated into traditional wastewater treatment processes [15]. 

Several studies have demonstrated the ability of different green microalgal species to remove both nutrients and HM from polluted media, in particular *Chlorella* sp., *Scenedesmus* sp., *Desmodesmus* sp. and *Chlamydomonas* sp. [16,17,18,19]. In addition to the employment of a low cost-media for microalgae cultivation, wastewater treatment utilizing cyanobacteria and green microalgae also offers the advantage of the concomitant production of added-value products from the biomass obtained during the process. Microalgal biomass extracts can be used in the field of energy, aquaculture, or even as food supplements [18,20,21]. As stated before, the capacity of plants to remove HM and nutrients is also widely recognized [22]. 

Coupling this remediation ability with biomass production of some species, such as *A. mauritanicus*, it is possible to create a circular economy model to produce a sustainable source of renewable energy [23]. Associations of microalgae and plants are often visible in hydroponic systems, as microalgae provides oxygen for plant root respiration from photosynthesis [24]. Only a few studies have focused on coupling the remediation abilities of microalgae and plants; therefore, there is still much to understand about this combination and whether it would result in an improved treatment. 

Recent studies on hydroponic systems coupling tomato plants with *Chlorella infusionum*, *Chlorella vulgaris* and *Scenedesmus quadricauda* [24,25] have reported a growth-promoting effect on all the involved organisms when co-cultivating. Furthermore, higher nutrient removal efficiency was also reported [15] for a co-cultivation of tomato plants and a mixed consortium of microalgae consisting mainly of *Chlorella* sp., along with *Scenedesmus* sp., *Synechocystis* sp. and *Spirulina* sp. compared to monoculture controls.

The aim of this work was to assess the feasibility of a combined approach using the green microalga *Desmodesmus* sp. and/or the cyanobacterium *Nostoc* sp. coupled with the hemicryptophyte *A. mauritanicus* for bioremediation of polluted waters with an excess of nutrients (nitrogen and phosphorus) and HM (copper and nickel).

## 2. Results

The water used as a culture medium in the present work was collected in the Fosso del Cavaliere river. This waterbody is monitored by the University of Rome Tor Vergata as it is located on the campus, and it is very polluted because it crosses a densely populated urban area and an industrial site. However, Cu and Ni were added, either alone or in combination because, despite its pollution, the water of the Fosso del Cavaliere was fortunately free of HM. Pre-treatment of the water involved filtration to remove any suspended solids and particulates that were naturally present. 

### 2.1. Preliminary Experiments with Microalgae

**Figure 1:**
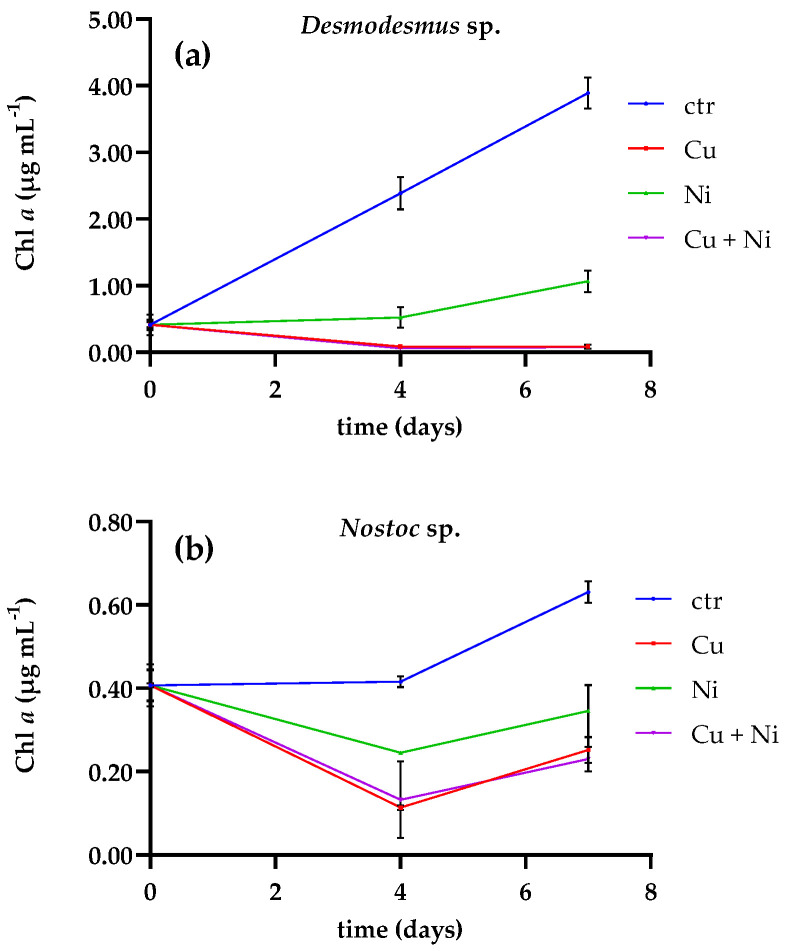
Time courses of the Chl *a* content (expressed in µg mL^-1^) in (**a**) *Desmodesmus* sp. and (**b**) *Nostoc* sp. in the absence (ctr) or presence of Cu, Ni and the mix of the two metals. Data are the mean values ± standard deviations (*p* < 0.05 vs. control).

The growth rates of *Desmodesmus* sp. and *Nostoc* sp. were significantly reduced in water containing Cu and the mixture of metals (µ < 0.01; *p* < 0.05) compared to the control (µ = 0.091 and 0.087 for *Desmodesmus* sp. and *Nostoc* sp., respectively). The chlorophyll *a* (Chl *a*) content in the control cultures of *Desmodesmus* sp. was more than 10-times higher than the cultures containing Cu (3.887 ± 0.235 and 0.086 ± 0.03 µg mL^−1^, respectively) after seven days (Figure 1a). 

In water containing a mixture of the two metals, the Chl *a* concentration decreased from 0.411 ± 0.077 µg mL^−1^ (day 0) to 0.079 ± 0.001 µg mL^−1^ (day 7), while those containing only Ni were the less affected, with a Chl *a* content of 1.065 ± 0.164 µg mL^−1^ at day 7. Similar to *Desmodesmus* sp., the growth of *Nostoc* sp. was more affected by Cu than Ni (*p* < 0.001). The Chl *a* content in the culture of *Nostoc* sp. exposed to Cu decreased from 0.406 ± 0.051 µg mL^−1^ to 0.250 ± 0.031 µg mL^−1^ (day 7). A decreasing trend was also shown in presence of both metals, with a final Chl *a* concentration of 0.230 ± 0.029 µg mL^−1^ (Figure 1b). 

**Figure 2:**
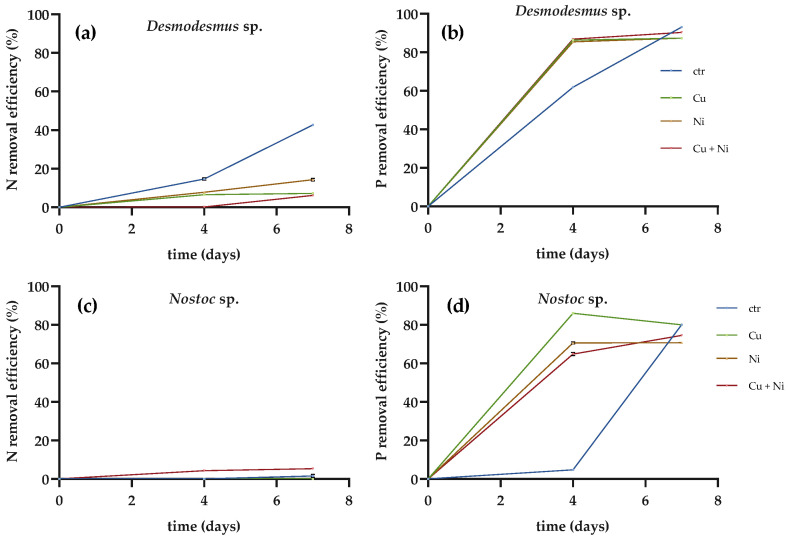
Removal efficiencies (%) of N and P by (**a**,**b**) *Desmodesmus* sp. and (**c**,**d**) *Nostoc* sp. in control cultures and in the presence of Cu, Ni and the mix of the two metals (data are the mean values ± standard deviations; differences were considered significant for *p* < 0.05, two-way ANOVA).

The N and P removal efficiencies (Figure 2) showed that the presence of metals reduced the nutrient removal capacities of both *Desmodesmus* sp. and *Nostoc* sp. However, the green microalga maintained some removal ability, although the results obtained in cultures grown in presence of metals were significantly lower than the controls (*p* < 0.001). 

N removal by *Desmodesmus* sp. (Figure 2a) decreased from 44% (control) to 5% in the presence of Cu, 13% in the presence of Ni and 8% with both metals. P removal (Figure 2b) was decreased from 75% (control) to 46% in Cu and 49% in Ni and 57% in both metals. *Nostoc* sp. had a low N removal ability (Figure 2c) under all conditions, while its P removal capacity (Figure 2d) was more affected by Ni than Cu (*p* < 0.001). 

The metal removal capacity (Table 1) was also evaluated and showed that, in seven days, *Desmodesmus* sp. had removed 67% of the initial Cu, resulting in a final concentration of 0.37 mg L^−1^. *Nostoc* sp. was unable to remove Cu from the water. *Desmodesmus* sp. was highly effective at removing Ni, with values more than 10 times those of *Nostoc* sp. (Table 1). In solution with both metals (0.89 mg L^−1^ Cu and 1.76 mg L^−1^ Ni), *Desmodesmus* sp. exhibited the highest removals for Cu (57%) and Ni (30%), with a residual concentration of 0.38 and 1.22 mg L^−1^, respectively. *Nostoc* sp. showed a Cu removal of 28% (residual content of 0.64 mg L^−1^) and a Ni removal of 11% (residual concentration of 1.56 mg L^−1^). 

### 2.2. Sequential Tests A. mauritanicus/Desmodesmus sp.

*Desmodesmus* sp., due to its metal resistance and uptake capacity of both nutrients and metals, was selected for a sequential test with *A. mauritanicus*. Preliminary tests established that *A. mauritanicus* and *Desmodesmus* sp. would not grow together because when *A. mauritanicus* plants were incubated in combination with *Desmodesmus* sp., the microalgae attached to the plant roots, and the plant leaves showed signs of bleaching; therefore, we decided to carry out a sequential test. First, *A. mauritanicus* was incubated in the test water for seven days, followed by a seven-day incubation with *Desmodesmus* sp. (a total of 14 days). The initial total N and total P concentrations were different from test 1, as the water was sampled on different occasions. *A. mauritanicus* showed no signs of stress in the presence of the tested concentrations of metal, and both the quantities of chlorophyll and their morphology did not change compared to the control (data not shown).

**Figure 3:**
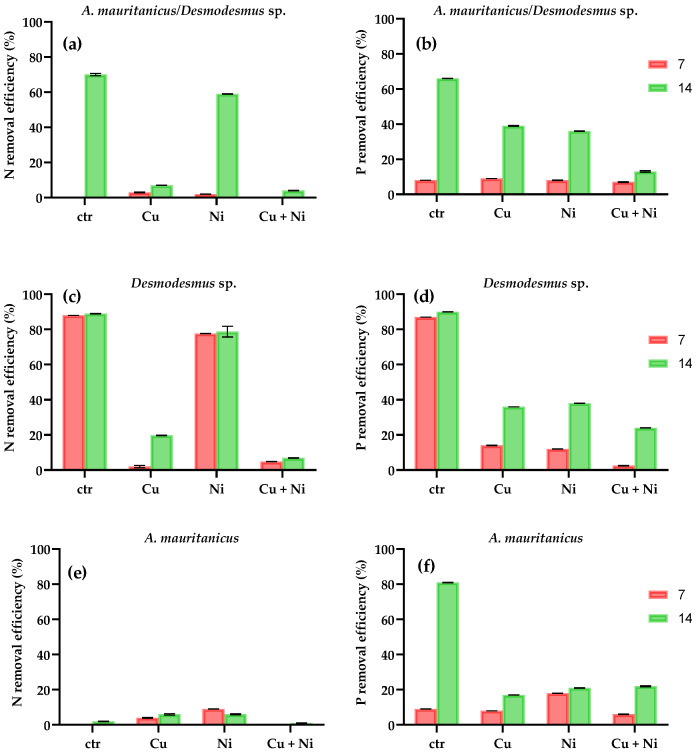
N and P removal efficiencies (expressed as percentage after 7 and 14 days of treatment) of Test 2 in: (**a**,**b**) *A. mauritanicus/Desmodesmus* sp., (**c**,**d**) *Desmodesmus* sp. and (**e**,**f**) *A. mauritanicus*. (Data are the mean values of triplicates ± standard deviations). Multiple comparisons test was used to analyze data along with two-way ANOVA with Tukey’s. Differences were considered significant at *p* < 0.05.

As in the first test, the results obtained (Figure 3) showed that the presence of metals affected the nutrient removal efficiencies of both *Desmodesmus* sp. and *A. mauritanicus* as single species and in the sequential approach. In the presence of Cu and both metals, the N removal efficiency by *A. mauritanicus/Desmodesmus* sp. was less than 7%, while, in the presence of Ni, the N removal efficiency was 59% with a residual content of 3.01 mg L^−1^ after 14 days (Figure 3a). The N removal was 70% when grown in the absence of metals (control). In the presence of Cu and Ni, the P removal was 39% and 36%, respectively (Figure 3b), which were not statistically different (*p* > 0.05), and was only 13% in the combined metal solution (five-times lower than in the control cultures). 

There were no significant differences between the sequential approach *A. mauritanicus/Desmodesmus* sp. and single species control cultures of *A. mauritanicus* (*p* > 0.05 and *p* = 0.9069). In the single-species test, *Desmodesmus* sp. Demonstrated a greater ability to remove N and P over *A. mauritanicus* (*p* < 0.0001). The N removal efficiency of *Desmodesmus* sp. alone (Figure 3c) was significantly lower in media containing Cu compared with Ni: after 14 days, N removal in the presence of Cu was 21% and 79% in Ni cultures and only 4% in the presence of both metals. 

Compared to the 90% of P removal in control cultures, the P removal efficiency of *Desmodesmus* sp. was reduced in the presence of metals: the effect of single metals was similar (36% of P removal in presence of Cu and 38% for Ni), while the presence of both metals reduced the P removal at only 24% (Figure 3d). After the 14-day incubation, the N removal efficiency of *A. mauritanicus* was around 6% in all the conditions tested (Figure 3e). The P removal efficiency after 14 days was 17% in the presence of Cu, 21% in the presence of Ni and 22% for both metals. The P removal efficiencies of *A. mauritanicus* control reached the 90% after 14 days (Figure 3f). 

**Figure 4:**
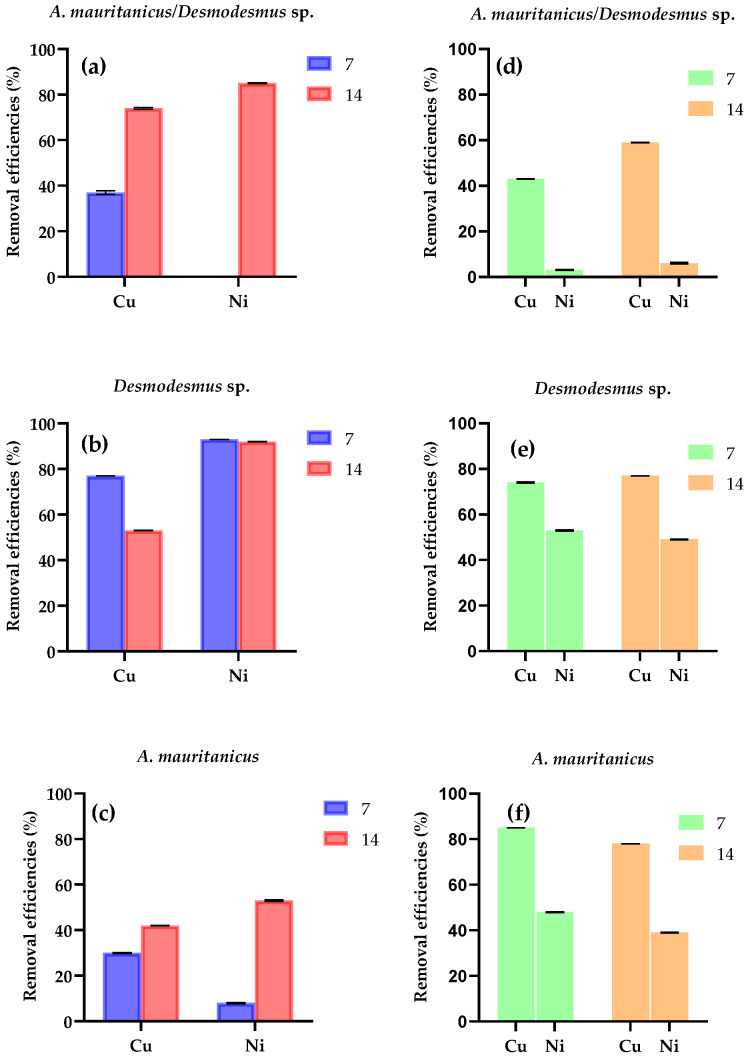
Cu and Ni removal efficiencies of sequential tests of (**a**–**c**) single metal solution and (**d**–**f**), mix solution of cu and Ni. Removal efficiency (expressed as percentage, %) reported after 7 and 14 days of treatment (data are the mean values of triplicates ± standard deviations). A multiple comparisons test was used to analyze data along with two-way ANOVA with Tukey’s. Differences were considered significant at *p* < 0.05.

The *A. mauritanicus*/*Desmodesmus* sp. results from Test 2 showed that, after 14 days, the metal removal efficiencies were 74% for Cu (giving a residual concentration of 0.29 mg L^−1^) and 85% for Ni (giving a residual concentration of 0.29 mg L^−1^) (Figure 4a). The Cu removal in the bi-metal solution was 59% with a residual concentration of 0.36 mg L^−1^ (Figure 4d). The removal efficiencies after 14 days in single species experiments were significantly higher for *Desmodesmus* sp. Than for the combination of *A. mauritanicus*/*Desmodesmus* sp. and *A. mauritanicus* alone (*p* < 0.001 and *p* = 0.00075). *Desmodesmus* sp. (Figure 4b) exhibited the highest Cu removal efficiency after 7 days (77%) with a residual content of 0.27 mg L^−1^; however, after 14 days, the overall Cu removal was reduced to 53%. 

This might be explained by a release of Cu in the aqueous medium by the green microalga, due to the reversible metal ion adsorption on the cell walls of microalgae [26]. The Ni removal efficiency of *Desmodesmus* sp. was higher than *A. mauritanicus*, showing a 93% of removal after 7 days that remained stable until the end of the test, while the *A. mauritanicus* removed 53% of the medium Ni, by day 14 (Figure 4c). In the bi-metal solution, the Cu and Ni removal by single cultures of *Desmodesmus* sp. and *A. mauritanicus* was always higher than that measured in the combined sequential system (Figure 4e,f). 

## 3. Discussion

The present work was a preliminary bioremediation test to evaluate the nutrient and metal removal capacity of two strains of microalgae, a metallophyte and a sequential alga-higher plant treatment. Few studies have focused on coupling the wastewater remediation abilities of plants and microalgae to understand if these combinations could improve treatment potentials [27]. According to the European Water Framework Directive (2000/60 EC) before discharging into water bodies, the total nitrogen (TN) and total phosphorus (TP) concentrations must be reduced to 15 mg L^−1^ and 2 mg L^−1^ for agglomerations of 10–100 thousand population equivalents (PE). For more sensitive areas (more than 100 thousand PE), the TN levels must be reduced to 10 mg L^−1^ and the TP must not exceed 1 mg L^−1^. 

A preliminary test was carried out to choose between the microalga, *Desmodesmus* sp., and a cyanobacterium, *Nostoc* sp., based on their metal and nutrient uptake capacity, to use in combination with *A. mauritanicus*. The growth rates of the green microalga and the cyanobacterium were significantly reduced by the presence of metals (*p* < 0.05), especially in the presence of Cu, either alone or in combination with Ni. 

The stress caused by the metals was revealed by the final Chl *a* content, which was 97% lower in the Cu culture compared to the control. Adverse effects of Cu and Ni on the growth rate of *Desmodesmus* sp. were previously reported by Rugnini et al. [28], however, to a lesser extent (a reduction of 85% of Chl *a* at 11.9 mg Cu L^−1^). Even if microalgae showed a drastic change in vitality, they were still able to remove metals. Metal ions are taken up by microalgal biomass in a two-stage process: an adsorption and bioaccumulation process. At first, the metal ions are passively adsorbed on the cell surface (of both living and non-living biomass) in a few seconds or minutes; then, the ions are transported slowly inside the cell membrane and are accumulated intracellularly. Bioaccumulation occurs only in living cells [18,26]. 

Thus, even if the microalgae vitality is reduced, they are still able to remove metals from solution by passive adsorption, and therefore the removal of Cu and Ni in *Desmodesmus* sp. was higher than 50% in this culture conditions. On the other hand, the lower growth rate of *Desmodesmus* sp. is the likely explanation of the lower N and P removal capacities obtained in cultures. The metal toxicity effect on growth could also explain why *Nostoc* sp. had even lower N and P removal efficiencies in the presence of metal. Similar findings of an inhibitory Ni effect limiting the nutrient uptake capacity of *C. vulgaris* were previously determined [29]. 

The N removal efficiency of *C. vulgaris* was affected at only high Ni concentrations, and P removal was far more sensitive to Ni. However, in this experiment, the effect of metals on P removal was lower than that on N removal. Previous studies, on the other hand, showed for *Desmodesmus* sp., a P removal efficiency between 94% and 100% in the presence of Cu and Ni singularly or in combination, and a strain of *Chlorella* sp. [30] exposed to different metals (Cr, Cu, Mn, Zn, Fe, Na and K) showed no effect on the nutrient uptake. Due to the significant effect of metals on the nutrient uptake rates for *Nostoc* sp., we decided to conduct the sequential study using *Desmodesmus* sp. with *A. mauritanicus*. As observed in test 1, the presence of metals significantly reduced the nutrient removal efficiency, in particular N (*p* < 0.05). 

The highest N removal in the *A. mauritanicus/Desmodesmus* sp. test occurred in the presence of Ni (59%), out of which only 2% was removed until day 7 by *A. mauritanicus.* Although *A. mauritanicus* is considered a metallophyte, it appears to be sensitive to Ni. Many studies have shown that HM interfere with the physiological processes of plants, one of which is nutrient absorption [31], in *Arabidopsis thaliana* L. [32] and *Silene vulgaris* (Moench) Garcke [33], Cu was shown to reduce the uptake of nitrogen, among other nutrients. Two-way ANOVA tests showed that, apart from the microalga and the plant, the N removal efficiency was dependent on which metal was present (*p* < 0.05) while the P removal was indifferent (*p* > 0.05). 

The highest Cu removal efficiency (74%) was obtained in the sequential treatment of *A. mauritanicus/Desmodesmus* sp., while the single species controls showed removals of less than 53%. A similar result was shown for a consortium between *Pistia stratiotes* and *Ankistrodesmus* sp. [34], which showed a higher efficiency than the results reported in our work, with a Cu removal of 90% after 72 h. Therefore, in this case, the sequential treatment *A. mauritanicus/Desmodesmus* sp. was more effective than the single species removing Cu. On the contrary, in the presence of Ni, the sequential test efficiency was lower than the single species.

In this study, the Ni removals were higher than those reported by Rugnini et al. [28] (12 days, initial Ni concentration of 1.9 mg L^−1^, removal rates of 30% for *Desmodesmus* sp. and 6% for *Chlorella* sp.). However, the same preferential cell-binding affinity of Cu over Ni in multi-metal solutions was reported. This preference could be explained by the stronger binding strength and larger ionic radius of Cu compared to Ni, as these will increase the covalent interaction between the metal ion and the ligands—in particular, the carboxylate groups of algal cell walls [18].

In the bi-metal solution tests, the single species resulted as more efficient compared to the sequential test (*p* < 0.05). Several studies have been performed to determine the remediation ability of microalgae and plants individually and yet there is a scarcity of information on the combined effects of plants/microalgae for pollutant removal. Numerous studies have reported on the algistatic or algicidal effects of plant allelochemicals [35], and some showed allelopathic substances of terrestrial plants in aquatic systems. 

Fifteen allelopathic substances in extracts of *Cyperus alternifolius* L. and *Canna generalis* L.H. Bailey & E.Z. Bailey have been determined, most of which inhibited *Microcystis aeruginosa* growth [36], while aqueous extracts of rice straw [37] are effective algicides. This could explain the low removal efficiency observed in the sequential test plants/algae carried out in this study when compared to the control. 

## 4. Materials and Methods 

### 4.1. Microalgal Strains

**Figure 5:**
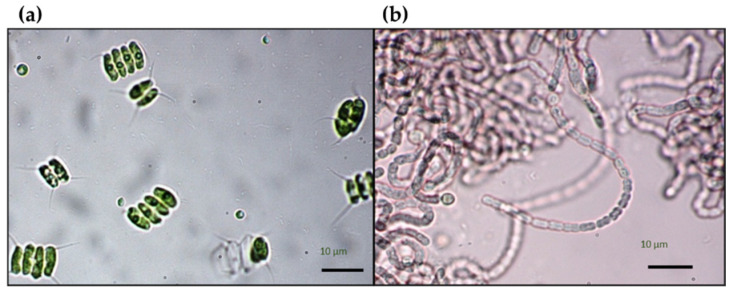
Light microscope (LM) images of the isolated strain of the (**a**) green microalga *Desmodesmus* sp. VRUC281 and (**b**) the cyanobacterium *Nostoc* sp. VRUC270 at 40×.

The strains selected for this study were the green microalga *Desmodesmus* sp. VRUC281 and the cyanobacterium *Nostoc* sp. VRUC270 (Figure 5). Both strains were isolated from a secondary sedimentation tank of a municipal wastewater treatment plant (WWTP) and belong to the culture collection of the Tor Vergata Rome University Culture collection (VRUC) [38]. Stock cultures were grown in BG11 and BG11_0_ culture media [39], in 175 cm^2^ aerated flasks, in a temperature and light controlled chamber at 18 ± 2 °C and irradiance of 12 ± 2 µmol photons^−1^ m^−2^ s^−1^, with a light/dark cycle of 12: 12 h.

### 4.2. Plant Materials 

**Figure 6:**
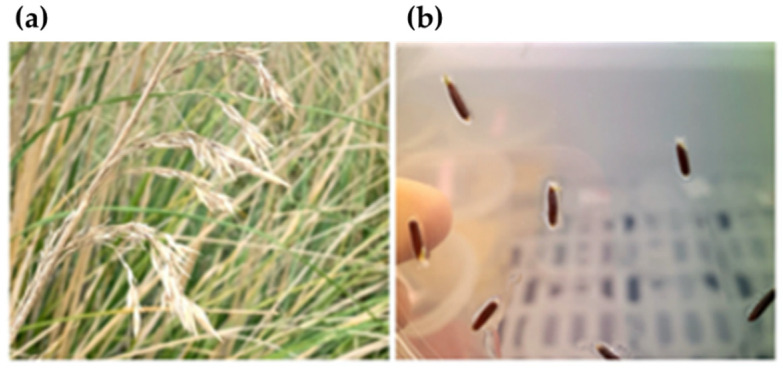
(**a**) Inflorescence of *A. mauritanicus*; and (**b**) in vitro sowing of *A. mauritanicus* kernels.

Seeds were obtained from plants grown in the Botanic Gardens of the University of Rome Tor Vergata, where a pilot phytoremediation system of approximately 143 m^2^ was installed for field tests of the uptake and degradation capacity of *A. mauritanicus*. Caryopses were collected in October 2019 and were deprived of glume and lemma before use. Subsequently, they were stored at 4 °C to maintain the viability and to synchronize the germination (Figure 6). For the sterilization procedures, the caryopses were exposed to 70% ethanol for 30 s, washed three times with autoclaved distilled water, followed by treatment with 15% (*w*/*v*) sodium hypochlorite and two drops of TWEEN^®^ 20 (SIGMA-ALDRICH, St Louis, MO-USA), kept on a gyratory shaker at 110 rpm for 1 h and then washed three times with sterile distilled water. 

*A. mauritanicus* caryopses were placed on micro-perforated floating rafts, inside a propylene box (90 × 140 mm), containing 250 mL of MS liquid medium [40] and 2% sucrose. The pH was adjusted to 5.6–5.8 before autoclaving at 121 °C for 20 min. Cultures were incubated in a growth chamber at 25 ± 2 °C under a 16/8 h light/dark-photoperiod by cool white led lamps at density of 30–40 µmol m^−2^ s^−1^. After four weeks, the plantlets were transferred to a new medium containing water of Fosso del Cavaliere river that had been previously membrane filtered (pore size 0.2 micron) and autoclaved. Before transfer, the roots were washed three times with distilled water.

### 4.3. Preliminary Test with Microalgae

In test 1, *Desmodesmus* sp. and *Nostoc* sp. strains were grown for seven days in water of Fosso del Cavaliere river (Rome, Italy, 41°50′55.2″ N 12°38′49.7″ E), pre-filtered with a Millipore^®^ Stericup^®^ filtration system and supplemented with copper (Cu), from copper sulphate (CuSO_4_·5H_2_O, Sigma-Aldrich reagent grade) or nickel (Ni), from nickel sulphate (NiSO_4_·7H_2_O, Sigma-Aldrich reagent grade), as single (1.15 mg Cu L^−1^, 1.86 mg Ni L^−1^ ) or bi-metal solutions (0.89 mg Cu L^−1^ + 1.76 mg Ni L^−1^). The HM concentrations used in the present work are the average of those detected in other metal polluted waterbodies we have studied. A control lacking both metals was also used. The growth was monitored through optical density (OD) and the Chl *a* content immediately after the inoculum (t_0_) and at days 4 and 7.

### 4.4. Sequential Test

Sequential treatments were used because, when eight-weeks-old *A. mauritanicus* plants (ranging from 100 to 120 mm in height) were incubated in combination with *Desmodesmus* sp., after 7 days, the microalgae had attached to the plant roots, and the plant leaves showed signs of bleaching (data not shown). Thus, we decided to perform the second experiment (test 2) using a sequential approach. Hadiyanto et al. [41] employed aquatic plants, water hyacinth (*Eichhornia crassipes*) and water lily (*Nymphaea* sp.) as well as the cyanobacterium *Spirulina* sp. to reduce the COD and nutrient content in palm oil mill effluent. In the first stage, the authors employed plants (3–8 days) and then the microalgae (15 days). Thereby, we planned our experiment similarly. 

**Figure 7:**
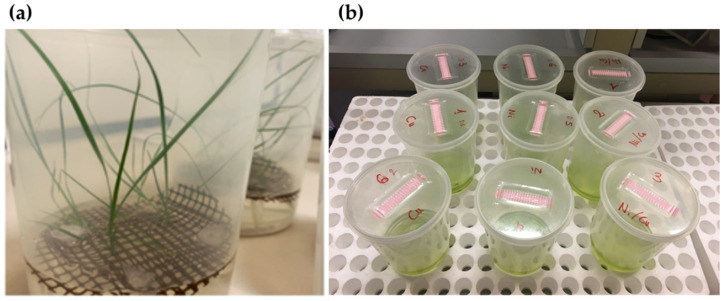
Sequential test employing (**a**) five plants of *A. mauritanicus* from day 0 to 7 and (**b**) *Desmodesmus* sp. from day 7 to 14, to evaluate the N, P, Cu and Ni removal.

During the first week (day 0 to 7), three plants of *A. mauritanicus* were grown in 250 mL solutions containing either Cu, Ni or a combination of both metals at the same concentration of test 1 (Figure 7a). After 7 days, the plants were removed and an inoculum of 15 mL of the green microalga *Desmodesmus* sp. was added to the culture medium and left for another week (day 7 to 14) (Figure 7b). The nutrient and metal removal efficiencies were evaluated by collecting 25 mL for each treatment, which was repeated at the beginning of the experiment and at 7 and 14 days for N and P analysis, while another 50 mL was collected for metal analysis on the same days. Three replicates with five plants in each box were used for each treatment. The control cultures were *A. mauritanicus* and *Desmodesmus* sp. singularly and combined in the water of Fosso del Cavaliere river containing no Cu and Ni. 

### 4.5. Evaluation of Growth 

Culture growth was monitored by measuring the optical densities at 560 (OD_560_) and 730 nm (OD_730_) with an ONDA-UV spectrophotometer for *Desmodesmus* sp. and *Nostoc* sp., respectively. The growth rates (μ) were calculated from a triplicate measure of OD of the cultures, according to Rugnini et al. [18].

The growth was also evaluated in terms of the variation in the chlorophyll *a* content. Pigment extraction was performed according to Wellburn and Lichtenthaler [42] in methanol at 95%. The Chl *a* concentration was calculated as follows (Equation (1)):

(1) $$Chl_{a}\left(\frac{\mu g}{mL}\right)=15.65\times A_{666}-7.34\times A_{653}.$$

### 4.6. Nutrient Analysis

Due to the intrinsic variability of nitrogen (N) and phosphorus (P) concentration in the river of Fosso del Cavaliere, the TN and TP were evaluated at the beginning of each test. The N and P removal efficiencies were determined by detecting the residual concentrations in the media, after sample filtration with Whatman paper filters (1.2 µm pores). The removal efficiencies were also determined after 4 and 7 days according to Langer and Hendrix [43]. The N and P removal efficiencies were calculated using Equations (2) and (3):

(2) $$N_{\%}=\frac{N_{0}-N_{t}}{N_{0}}$$

where *N_0_* is the initial concentration of *N* and *N_t_* is the concentration of *N* after *t* days of cultivation [31,32].

(3) $$P_{\%}=\frac{P_{0}-P_{t}}{P_{0}}$$

where *P_0_* is the initial *P* concentration and *P_t_* is the concentration of *P* after *t* days of cultivation.

### 4.7. Metal Analysis

The concentrations of Cu and Ni were analyzed by inductively coupled plasma atomic emission spectroscopy (ICP-OES). The Cu and Ni removal efficiencies were determined by detecting the residual concentrations in the media after 50 mL of sample filtration with Whatman paper filters (1.2 µm pores) at days 4 and 7. Before the analyses, the samples were acidified to pH < 2 with ultrapure nitric acid (APAT CNR IRSA 3010A Man 29 2003 + APAT CNR IRSA 3020 Man29 2003).

The Cu and Ni removal efficiencies (Removal, %) were evaluated according to Zhou et al. [44] (Equation (4)):

(4) $$Removal=\frac{\left(C_{0}-C_{f}\right)\times 100}{C_{0}}$$

where *C*_0_ is the initial concentration of either Cu or Ni (mg L^−1^) and *C_f_* is the final concentration. All experiments were performed in triplicates. 

### 4.8. Statistical Analysis 

All results are expressed as the mean +/- standard deviation from three different replicates. Data were analyzed using the GraphPad Prism software, version 9.0.1 (San Diego, CA, USA). To compare the differences in the growth curves of *Desmodesmus* sp. and *Nostoc* sp. in the presence of metals (Figure 1), an unpaired *t*-test was used. To analyze the influence of metals in the N and P removal efficiencies (Figure 2) a two-way ANOVA with Tukey’s multiple comparisons test was employed. A multiple comparisons test was used to analyze the data of test 2 along with two-way ANOVA with Tukey’s (Figure 3 and Figure 4). Differences were considered significant at *p* < 0.05. 

## 5. Conclusions

Both microalgae and plants exhibited a high potential for the remediation of polluted waters from excess nutrients and metals. The present work evaluated the feasibility of a sequential approach exploiting the hemicryptophyte *A. mauritanicus*, the green microalga *Desmodesmus* sp. VRUC281 and the cyanobacterium *Nostoc* sp. VRUC270 for N, P and metal (Cu and Ni) removal. The presence of metals caused a reduction in growth and in the nutrient removal ability. 

*Nostoc* sp. was more sensitive to the presence of metals; therefore, *Desmodesmus* sp. was chosen for sequential tests using plants and microalgae. However, the green microalga alone was able to remove more nutrients compared to the sequential plant/microalga treatment. The sequential approach had a higher metal removal capacity in the presence of singular metals: up to 74% for Cu and 85% for Ni in 14 days. In the bi-metal solutions, the removal rates were lower with a preferential affinity for Cu over Ni (43% for Cu and 6% for Ni). 

The removal rates were higher in the single species controls, indicating a negative effect possibly as a result of allelopathic phenomena between plants and algae. The plant and the microalga selected for this study presented better removal abilities for nutrients and metals when used singularly compared to the sequential approach. Further studies are necessary to define the mechanisms underlying these results as well as to exploit new combination of plants and microalgae species.

## Figures and Tables

**Table 1:** Metal removal efficiencies of *Desmodesmus* sp. and *Nostoc* sp. evaluated in Test 1, after 7 days of metal treatment (data are the mean values of triplicates), where C_f_ (mg L^−1^) represents the final concentration (data are the mean values ± standard deviations) and Removal (%) the removal efficiency of metal present in solution (data are the mean values ± standard deviations; differences were considered significant for *p* < 0.05, two-way ANOVA).

	Initial [Cu] 1.15 mg L^−1^		Initial [Ni] 1.86 mg L^−1^		Mix 0.89 mgCu L^−1^ + 1.76 mgNi L^−1^			
	C_f_ (mg L^−1^)	Removal (%)	C_f_ (mg L^−1^)	Removal (%)	C_f_ (mg L^−1^)		Removal (%)	
					Cu	Ni	Cu	Ni
*Desmodesmus* sp.	0.37 ± 0.04	67%	0.33 ± 0.07	82%	0.38 ± 0.09	1.22 ± 0.31	57%	30%
*Nostoc* sp.	1.70 ± 0.41	n.d.	0.73 ± 0.09	61%	0.63 ± 0.03	1.56 ± 0.19	28%	11%
